# Differences in the Clinical and Hematological Characteristics of COVID-19 Patients with and without Type 2 Diabetes

**DOI:** 10.1155/2020/1038585

**Published:** 2020-12-02

**Authors:** Yujing Sun, Ruxing Zhao, Zhao Hu, Weili Wang, Shouyu Wang, Ling Gao, Jianchun Fei, Xiangdong Jian, Yu Li, Huizhen Zheng, Xinguo Hou, Li Chen

**Affiliations:** ^1^Department of Endocrinology, Qilu Hospital, Cheeloo College of Medicine, Shandong University, Jinan 250012, China; ^2^Institute of Endocrine and Metabolic Diseases of Shandong University, Jinan 250012, China; ^3^Jinan Clinical Research Center for Endocrine and Metabolic Diseases, 250012, China; ^4^Department of Nephrology, Qilu Hospital, Cheeloo college of Medicine, Shandong University, Jinan 250012, China; ^5^Department of Endocrinology, Renmin Hospital of Wuhan University, Wuhan 430060, China; ^6^Department of Anesthesiology, Qilu Hospital, Cheeloo College of Medicine, Shandong University, Jinan 250012, China; ^7^Department of Emergency, Qilu Hospital, Cheeloo College of Medicine, Shandong University, Jinan 250012, China; ^8^Department of Respiratory, Qilu Hospital, Cheeloo College of Medicine, Shandong University, Jinan 250012, China

## Abstract

**Objective:**

To examine whether comorbidity with type 2 diabetes (T2D) affects the clinical and hematological parameters of coronavirus disease 2019 (COVID-19) patients.

**Methods:**

We retrospectively investigated the clinical, imaging, and laboratory characteristics of patients with confirmed COVID-19 who were hospitalized from January 30, 2020 to March 17, 2020, at the Renmin Hospital of Wuhan University. A detailed clinical record was kept for each subject, including the medical history of COVID-19 and physical and laboratory examinations. A total of 164 subjects were eligible for the study, among which 40 patients were comorbid with T2D. Further analysis was conducted in two subcohorts of sex- and age-matched patients with and without T2D to identify hematological and biochemical differences. The laboratory tests, including routine blood tests, serum biochemistry, and coagulation function, were performed upon admission.

**Results:**

The two groups showed no significant differences in baseline parameters, including age, sex, chest X-ray, or computed tomography (CT) findings, upon admission. However, patients with T2D showed an increased incidence of diarrhea. T2D patients required more recovery time from pneumonia, as shown by follow-up CT findings, which might contribute to the prolonged hospitalization. Comorbidity with T2D also increased risk of secondary bacterial infection during COVID-19. The T2D group had significantly higher white blood cell and neutrophil counts compared with the nondiabetic group, but T2D patients suffered from more severe lymphocytopenia and inflammation (*P* < 0.05). Most biochemical parameters showed no significant differences between the two groups (*P* > 0.05). However, patients with T2D seemed to have a significantly higher risk of developing hyperlactatemia, hyponatremia, and hypocalcemia.

**Conclusions:**

COVID-19 patients comorbid with T2D demonstrated distinguishing clinical features and hematological parameters during the infection. It is necessary to develop a different clinical severity scoring system for COVID-19 patients with T2D. This study may provide helpful clues for the assessment and management of COVID-19 in T2D patients.

## 1. Introduction

In December 2019, several hospitals in Wuhan City, Hubei Province, found multiple cases of unexplained pneumonia with a history of exposure to the South China Seafood Market [1, 2]. On February 11, 2020, the World Health Organization named the disease: coronavirus disease 2019 (COVID-19) [3]. COVID-19, which is caused by a beta-coronavirus, spread widely, and the number of confirmed cases increased daily. The characteristics of this new type of coronavirus pneumonia are significantly different from the characteristics of previous coronavirus diseases, such as SARS and MERS [4]. COVID-19 primarily manifests as viral pneumonia after infection, and a wide range of the population is susceptible to it. Clinical features of COVID-19 patients often include fever, dry cough, sputum, runny nose [5], fatigue, or poor breathing [6]. Lung computed tomography (CT) imaging typically shows ground-glass changes affecting both lungs [7].

COVID-19 has the potential to spread with influenza-related mortality, and comorbidity with diabetes may represent an important risk factor for adverse outcomes [8, 9]. One report showed that among critically ill adult patients, 21 (40%) patients had chronic diseases, with diabetes accounting for 17%. Another recent study showed that glucose metabolism was the basis of the influenza viral infection, leading to a fatal inflammatory reaction [10]. The same study showed that the mortality rate of diabetic patients was higher than that of nondiabetic patients [11]. Therefore, COVID-19 patients with diabetes comorbidity may have unique disease manifestations [12–14] and laboratory findings, as well as different prognoses, compared with patients without comorbities [15].

Identifying and distinguishing diabetic COVID-19 patients may help to avoid misdiagnosis and improper treatment and may improve prognostic evaluation by clinicians [16]. In this pilot study, based on the retrospective clinical data, we focused on the clinical and hematological characteristics of patients infected with COVID-19 with and without comorbid type 2 diabetes (T2D). Our goal was to determine whether comorbidity with T2D had unique influences on COVID-19 patients.

## 2. Materials and Methods

### 2.1. Subjects and Enrollment

We retrospectively investigated the clinical, imaging, and laboratory characteristics of patients with confirmed COVID-19 who met the inclusion criteria and did not meet the exclusion criteria and were consecutively enrolled at the Renmin Hospital of Wuhan University from January 30, 2020 to March 17, 2020. Renmin Hospital of Wuhan University is a government-designated hospital that unconditionally accepts nucleic-acid positive confirmed patients. A total of 164 subjects were eligible and enrolled in this study of clinical characteristics, among which 40 patients also had T2D, while the rest were nondiabetic. The investigation for this study subset of patients represented a consecutive cohort study of diabetic and nondiabetic populations in a community. Consequently, the differences in morbidity, symptom severity classification, signs and symptoms at admission, comorbidities, period of hospitalization, and disease course between diabetic and nondiabetic populations could be compared and analyzed. A detailed clinical record was kept for each subject, including COVID-19 medical history, physical examination results, and biochemical indicators. Laboratory examinations and further analyses were improved and conducted in two subcohorts of sex- and age-matched patients both with and without type 2 diabetes (referred to as the T2D groups) and nontype 2 diabetes (referred as the NDM groups), respectively, both *n* = 40, in search of hematological and biochemical differences. To ensure the accuracy of the clinical data, two researchers reviewed the electronic medical records independently. Patients' medical charts were reviewed and analyzed in a blinded manner.

**Figure 1:**
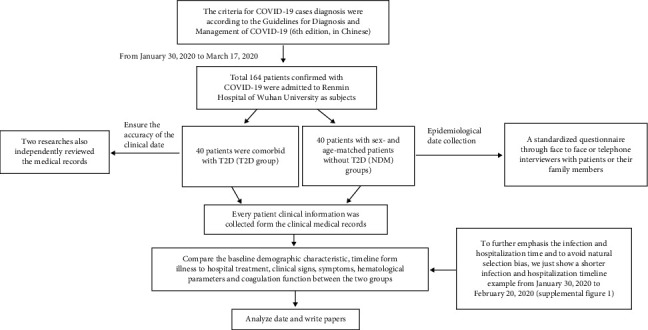


COVID-19 diagnoses were confirmed through epidemiological history, clinical manifestations, CT findings, and positive nucleic acid tests. Patients with confirmed or suspected type 1 diabetes, or other special types of diabetes, were excluded from the study. Patients with an uncertain diagnosis of either COVID-19 or type 2 diabetes were also excluded from the study. The data collection forms are shown in Figure 1.

### 2.2. Clinical Variables

Detailed clinical data were collected, including age, sex, exposure history, comorbid conditions, symptoms, and laboratory results. Well-trained attending physicians were responsible for the diagnostic procedures, interpretation of laboratory analyses, and treatment decision during the patients' in-hospital stays. We retrospectively evaluated the medical history, as well as the physical, hematological, biochemical, radiological, and microbiological examination results. Epidemiological, clinical, laboratory, and radiological characteristics and treatments, as well as outcome data, were obtained from electronic medical records. The data collection forms were reviewed independently by two researchers. The participants' medical records were reviewed for results of previously performed disease tests in a stable phase, symptoms, and findings from the physical examination performed at admission, including results from chest CT and laboratory tests. Patients' medical charts were reviewed and analyzed in a blinded manner.

According to the Guidelines for Diagnosis and Management of COVID-19 (6th edition, in Chinese) released by the National Health Commission of China, the clinical classifications of COVID-19 [17] are as follows:

Mild cases: the clinical symptoms are mild, and no pneumonia manifestation can be found in imaging.

Moderate cases: patients have symptoms like fever and respiratory tract symptoms, and pneumonia manifestation can be seen in imaging.

Severe cases: meeting any of the following: respiratory distress, *respiratory* *rates* ≥ 30 *breaths*/min, the *SpO*2 ≤ 93% at a rest state; *PaO*2/*FIO*2 *ratio* ≤ 300, and patients with >50% lesions progression within 24 to 48 hours in pulmonary imaging should be treated as severe cases.

Critical ill cases: meeting any of the following: respiratory failure occurs and mechanical ventilation is required, shock occurs, and complicated with other organ failure that requires monitoring and treatment in ICU.

Secondary bacterial infection was diagnosed depending on positive findings of the microbiological examination and significantly increased procalcitonin (*procalcitonin* > 0.1 *ng*/*mL*).

All the following criteria had to be met for hospital discharge, consistent with previous publication 18: (1) normal temperature lasting longer than 3 days, (2) resolved respiratory symptoms, (3) substantially improved acute exudative lesions on chest computed tomography (CT) images, and (4) 2 consecutively negative RT-PCR test results separated by at least 1 day [18].

### 2.3. Statistical Analysis

Results were expressed as mean ± standard deviation (SD) or median (interquartile range, IQR). The classification variable was represented as a count (%). The differences between the two groups were determined by a paired *t* test unless the data were not normally distributed, in which case a Mann–Whitney *U* test was used instead. A chi-square goodness-of-fit test was used for the rate comparison. All statistical analyses were performed using SPSS (v.18.0; SPSS Inc., Chicago, IL, USA). *P* value ≤0.05 was considered to indicate statistical significance.

## 3. Results

### 3.1. Baseline Demographic Characteristics of COVID-19 Patients with and without T2D

There were no significant differences between the two groups regarding the demographic characteristics of age, sex, or signs and symptoms at admission. Symptoms refer to the clinical manifestations observed at the onset of disease. However, the clinical classification showed significant difference between the two groups. Nearly half of the NDM patients were categorized as mild type (48.78%), while only 12 of 40 patients with T2D (30.00%) were mild (*P* = 0.03). In severe COVID cases, 9 patients with T2D (22.50%) were categorized as severe compared with just 7.31% in patients without T2D (*P* = 0.01). One patient with T2D (2.50%) was categorized as critically ill, while only 0.6% of patients were critically ill in the groups without T2D (*P* ≤ 0.001). Our study indicates that COVID-19 patients comorbid with diabetes have a higher risk of developing severe and critical COVID-19 (Table 1).

### 3.2. Timeline from Illness to Hospital Treatment

All 80 patients reported that symptoms lasted for 5–30 days, and the median numbers of hospitalization days for the T2D and without T2D groups were 21.14 [6–37] and 23.49 (7–44) days, respectively (data represented as median [IQR], *P* = 0.04). Patients with diabetes thus had a longer hospitalization time (Table 1). To emphasize further the infection and hospitalization timeline and to avoid natural selection bias, we also included a shorter infection and hospitalization timeline example utilizing the data from January 30, 2020 to February 10, 2020. Representative data from 39 patients hospitalized with COVID-19 are shown in Supplemental Figure 1.

### 3.3. Clinical Signs and Symptoms of COVID-19 Patients with and without T2D

The differences in clinical signs and symptoms of patients infected with COVID-19 were not statistically significant between the two groups with regard to fever, cough, shortness of breath, muscle ache, headache, mental disorder symptoms, sore throat, nausea, and vomiting. However, patients with T2D showed an increased incidence of diarrhea compared with NDM patients (32.50% vs. 12.50%, *P* = 0.03). The rate of the secondary bacterial infection of T2D patients also demonstrated nominal significance compared with the rate shown by patients without T2D (32.50% vs. 15.00%, *P* = 0.048). Chest X-rays and CT findings of pulmonary lesions in patients with and without T2D exhibited no significant differences at admission (Table 2). However, COVID-19 patients with T2D showed slower lung lesion recovery during follow-up after hospitalization (Supplemental Figure 2). This feature might explain the prolonged hospitalization time of T2D patients.

### 3.4. Hematological Parameters of COVID-19 Patients with and without T2D

Patients with COVID-19 often had anemia, lymphocytopenia, and increased monocytes, but there were no significant differences among red blood cell count (×109/L) 4.04 (2.19–5.02) vs. 4.14 (2.78–5.56); data represented as median (IQR), *P* = 0.51; hemoglobin (g/L), 124.6 (91–161) vs. 128.8 (88–165), *P* = 0.28; lymphocyte (×10^9^/L), 1.43 (0.48–3.42) vs. 1.22 (0.33–3.48), *P* = 0.18; and monocyte count (×10^9^/L), 0.50 (0.1–1.36) vs. 0.52 (0.11–1.2), *P* = 0.76, between NDM and T2D patients (Table 3). There were also no differences in the incidence of patients with decreased red blood cell count (47.50% vs. 42.50%, *P* = 0.65), hemoglobin (40.00% vs. 30.00%, *P* = 0.35), lymphocytes (42.50% vs. 45.00%, *P* = 0.82), and increased monocytes (15% vs. 22.5% *P* = 0.39) between the NDM and T2D groups (Table 3). Interestingly, the absolute numbers for white blood cell counts (WBC, ×10^9^/L, data shown as median [IQR], 7.22 [3.03–20.05] vs. 5.32 [2.56–9.39], *P* = 0.01) and neutrophil counts were abnormally higher in T2D patients compared with NDM patients (×10^9^/L, 5.41 [1.63–17.66] vs. 3.28 [1.37–6.46] *P* < 0.01). The incidence of increased WBCs and neutrophils was also higher in the T2D group compared to the NMD group (WBCs, 15.00% vs. 0.00%, *P* = 0.03; neutrophils, 25.00% vs. 5.00%, *P* = 0.01). Despite this, most cases of increased WBCs and neutrophils did not involve secondary bacterial infection. The T2D group had more cases of increased serum C reactive protein (CRP) levels compared with the NMD group (62.5% vs. 50%), but the difference was not statistically significant (*P* = 0.26, *P* > 0.05). The average serum CRP levels in T2D patients were higher than those in the NDM group (51.44 vs. 25.11, mg/L) and showed significant difference (*P* = 0.024, *P* < 0.05) (Table 3).

We also examined the liver function, but there were no significant differences among average levels of serum aspartate aminotransferase (AST, 38.00 vs. 37.12, U/L, *P* = 0.22), alanine aminotransferase (ALT, 32.20 vs. 26.73, U/L, *P* =0.72), alkaline phosphatase (ALP, 78.3 vs. 72.20, U/L, *P* = 0.63), and *γ*-glutamyltransferase (GGT, 43.6 vs. 43.2, U/L, *P* = 0.37). Patients with COVID-19 often demonstrated decreased levels of serum total protein (TP) and albumin. The TP level in 25 patients across both groups was lower than the normal range. Albumin levels in 25 patients (62.50%) without diabetes and 23 patients (57.50%) with diabetes were lower than normal (85 g/L), but the differences between the groups were not statistically significant (*P* = 0.08). The ALB/GLB ratio was lower than normal in three patients (7.50%) without diabetes and seven patients (17.50%) with diabetes. The ALB/GLB ratio was also significantly different between T2D and NDM patients (*P* = 0.0067, *P* < 0.01) (Table 3).

We also analyzed biochemical parameters, including blood glucose, lactate, blood electrolyte concentration, anion gap, and osmotic pressure. The results showed that the blood glucose level of 40 patients (100%) with diabetes exceeded the normal range. The lactate level of 11 NDM patients (27.50%) and 25 T2D patients (62.50%) exceeded the normal range. The two groups showed significant difference (*P* = 0.02) in lactate levels. The incidence of hyponatremia was in the normal range in four NDM patients (10.00%) and 10 T2D patients (25.00%). The two groups differed significantly (*P* = 0.01) in sodium ion levels. Hypocalcemia was present in 11 patients (27.50%) without diabetes and 22 patients (55.00%) with diabetes. The two groups differed significantly (*P* = 0.04) in calcium ion levels. The anion gap (AGPK) level in three patients (7.50%) without diabetes and six patients (15.00%) with diabetes was lower than the normal range. The osmolarity level in eight patients (20.00%) without diabetes and eight patients (20.00%) with diabetes was lower than the normal range. Overall, there were no significant differences between the two groups (*P* > 0.05) for any of these biochemical parameters (Table 3).

We also analyzed the blood lipid concentration differences between the two groups. The total cholesterol (TCH) levels of one patient (2.50%) without diabetes and two patients with T2D (5.00%) were higher than normal. The level of TG in 7 patients (17.50%) without diabetes and 11 patients with T2D (27.50%) was higher than the normal range. The level of low-density lipoprotein (LDL) was lower than the normal range (≥1) in 17 patients (42.50%) without diabetes and 20 patients (50.00%) with T2D. The level of high-density lipoprotein (HDL) was higher than the normal range in two patients (5.00%) without diabetes and five patients (12.50%) with T2D, but there was no significant difference between the two groups (*P* > 0.05) in any of these parameters (Table 3).

### 3.5. Coagulation Function of COVID-19 Patients with and without T2D

We also analyzed coagulation function differences between T2D and NDM patients. The fibrinogen level increased in 15 NDM patients (38.46%) and 16 T2D patients (53.30%). The level of D-dimer was increased in 24 NDM patients (61.54%) and 22 T2D patients (73.33%). The level of fibrinogen degradation products was increased in 14 NDM patients (35.90%) and 8 T2D patients (26.67%). No statistical difference between the groups was present (Table 4).

## 4. Discussion

The ongoing COVID-19 pandemic, caused by a novel coronavirus called severe acute respiratory syndrome coronavirus 2 (SARS-CoV-2), is now a global crisis [19, 20]. Although SARS-COV-2 only produces mild flu-like symptoms in the majority of patients affected, the virus may lead to severe or even lethal complications, such as acute respiratory distress syndrome and multiorgan dysfunction. T2D is documented to be a common comorbidity with COVID-19 [14, 21]. Studies show that 20%–50% of patients in the current coronavirus COVID-19 pandemic have diabetes [22, 23]. An increasing number of studies have demonstrated the clinical and laboratory characteristics of COVID-19 in diabetic patients [22]. The latest data from China show that once a patient with diabetes is diagnosed with COVID-19, the mortality rate is eightfold higher than that of nondiabetic patients [24]. Some studies even suggest that a history of diabetes and fasting blood glucose values are predictors of mortality and morbidity [25–27]. Since the beginning of the COVID-19 pandemic, increasing numbers of clinical researchers have focused on diabetes due to the poor prognosis common in this patient subgroup [28].

As shown in our study, COVID-19 patients with diabetes occupy a larger proportion of patients classified as critically ill. Additionally, our timeline was based on the longitudinal observation of a natural cohort of 164 hospitalized subjects without artificial selection during the same period. Therefore, these data could indicate that COVID-19 patients comorbid with diabetes have a higher risk of developing into severe cases and, furthermore, into critically ill cases. This cohort also revealed a prolonged hospitalization time for T2D patients compared with NDM subjects in a comparatively unbiased natural population. It is worth noting that the discharge criteria in the designated hospital include the final clearance of the virus (confirmed by two consecutive negative RT-PCR test results). The hospitalization timeline reflected the intact process from symptom onset to clearance of the virus. Therefore, our data provided the representative natural history of COVID-19 development in both NDM and T2D patients. Interestingly, although the chest CT images of COVID-19 patients with diabetes at admission showed no difference between the NDM and T2D patients, the chest CT findings obtained during follow-up observation revealed that COVID-19 patients with T2D had slower lung lesion recovery rates during management after hospitalization. Taken together, these data revealed that comorbidity with T2D was characterized by delayed viral clearance and more persistent pulmonary inflammation.

While there was no statistically significant difference in the typical clinical signs and symptoms of COVID-19 upon admission, the incidence of diarrhea at admission was significantly different between the two groups. Some studies found that dysregulation of autonomic nerves and loss of healthy gut flora were the common causes of diarrhea in diabetic patients [29, 30]. Diabetic patients are prone to neurodegeneration, which may cause dysfunction of sympathetic and vagal nerves, leading to gastrointestinal peristalsis, which in turn results in gastroparesis, diarrhea, or constipation [31]. In the case of long-term high blood glucose, harmful bacteria in the intestine may grow in population, which may lead to disorder of the bacterial community structure and digestive disorders [32]. It may be important to supplement T2D patient diets with probiotics to maintain the balance of intestinal flora.

In our study, we also found that the rate of secondary bacterial infection is higher for COVID-19 patients with diabetes. Due to abnormal blood glucose metabolism in diabetic patients, immune cells and immune factors are dysregulated. This facilitates various infections; some patients have fungal infections and tuberculosis [33]. Early recognition of secondary infection and antibiotics may be preferred in T2D patients. Poor blood glucose control increases the chances of secondary infection [34]. Therefore, strict control of blood glucose is essential for the treatment of diabetic COVID-19 patients.

The coronavirus infection may cause autoimmune hemolysis, leading to viral anemia, even though there was no significant difference between the two groups [35]. Anemia in COVID-19-infected people may be related to nutritional deficiencies, medical treatment, bone marrow infiltrating diseases, or acute or chronic blood loss. After the COVID-19 infection, WBC and neutrophil counts were higher in the T2D group than in the NDM group. Although the underlying mechanism is not understood, our study indicates that these abnormal increases in WBC and neutrophil count (NEUT) were not associated with the bacterial infection. A previous report found that NEUT was related to cytokine storm induced by COVID-19 invasion and caused sustained inflammatory response [36]. WBC and NEUT levels may be related to the underestimation of severity in patients with diabetes, even in the absence of signs and symptoms. Patients with type 2 diabetes seem to have a significantly higher risk of developing hyperlactatemia. Hyperlactatemia may be caused by tissue hypoxia or metabolic disorders [37] and can also result from increased or accelerated aerobic glycolysis during the stress response [38]. The significance of the lactic acid level may be more of an exclusionary parameter. That is, the increase of lactate may indicate the presence of either tissue hypoxia or metabolic disorders.

In addition to hyperlactatemia, T2D patients seem to have a significantly higher risk of developing hyponatremia and hypocalcemia. Given these results, it is important to closely monitor the electrolytes in diabetic patients during management and follow-up. Dietary supplementation with calcium and vitamin D may be helpful. There are no effective drugs for the treatment of patients with COVID-19 [39]. Vaccines are still under development or in clinical trials and have not yet been applied to the overall population. Therefore, supportive care is of the utmost importance in COVID-19 management.

Our study provides useful clues and adds to increasing evidence about the clinical and hematological characteristics of diabetic COVID-19 patients. However, as a retrospective pilot study, the revealed differences do not provide adequate data to explain the mechanisms underlying our observations. Second, some data were unavailable at the time of analysis due to the rapid emergence of this infectious disease. Third, because of our study's limited number of patients and single-centered design, our data need to be further verified by larger samples and multicenter cohorts.

In conclusion, COVID-19 patients comorbid with T2D demonstrated distinguishing clinical features and hematological parameters during infection. It is necessary to develop a different clinical severity scoring system for patients with T2D who become infected with COVID-19. This study may provide helpful clues for the assessment and management of COVID-19 in such patients. We hope this pilot study may provide reference materials for more efficient supportive care and management of COVID-19.

## Figures and Tables

**Table 1:** Baseline characteristics of patients with and without T2D infected with COVID-19.

Variables	NDM group (*n* = 40)	T2D group (*n* = 40)	*P* value
Age (year)			
Age median (IQR), years	59.70 (30-86)	60.45 (38-88)	0.83
Sex			
Female, *n* (%)	21/40 (52.50%)	21/40 (52.50%)	1.00
Clinical classification			
Mild type, *n* (%)	80/164 (48.78%)	12/40 (30.00%)	0.03
Moderate type, *n* (%)	71/164 (43.29%) (43.29%)	18/40 (45.00%)	0.84
Severe cases, *n* (%)	12/164 (7.31%)	9/40 (22.50%)	0.01
Critically ill type, *n* (%)	1/164 (0.60%)	1/40 (2.50%)	≤0.001
Comorbidities at admission			
Cardiovascular and cerebrovascular, *n* (%)	18/40 (45.00%)	20/40 (50.00%)	0.65
Digestive system disease, *n* (%)	2/40 (5.00%)	1/40 (2.50%)	0.91
Respiratory system diseases, *n* (%)	3/40 (0.75%)	2/40 (5.00%)	0.98
Nervous system diseases, *n* (%)	1/40 (2.50%)	1/40 (2.50%)	1.00
Malignant tumor, *n* (%)	0/40 (0.00%)	1/40 (2.50%)	0.91
Chronic kidney disease, *n* (%)	6/40 (15.00%)	5/40 (12.50%)	0.56
Chronic liver disease, *n* (%)	1/40 (2.50%)	1/40 (2.50%)	1.00
Chronic obstructive pulmonary disease, *n* (%)	1/40 (2.50%)	1/40 (2.50%)	1.00
HIV infection, *n* (%)	0/40 (0.00%)	0/40 (0.00%)	1.00

*P* values indicate differences between T2D patients and NDM patients. *P* < .05 was considered statistically significant.

**Table 2:** Clinical characteristics of patients with and without T2D infected with COVID-19.

Variables	NDM group (*n* = 40)	T2D group (*n* = 40)	*P* value
Signs and symptoms at admission and treatment			
Fever, *n* (%)	36 (90.00%)	40 (100.00%)	0.13
Cough, *n* (%)	33 (82.50%)	35 (87.50%)	0.77
Shortness of breath, *n* (%)	16 (40.00%)	17 (42.50%)	0.10
Muscleache, *n* (%)	20 (50.00%)	16 (40.00%)	0.55
Headache and mental disorder symptoms, *n* (%)	12 (30.00%)	10 (25.00%)	0.79
Sore throat, *n* (%)	13 (32.50%)	12 (30.00%)	0.89
Diarrhoea, *n* (%)	5 (12.50%)	13 (32.50%)	0.03∗
Nausea and vomiting, *n* (%)	7 (17.50%)	9 (22.50%)	0.80
More than one sign or symptom, *n* (%)	32 (80.00%)	33 (82.50%)	0.98
Rate of secondary bacterial infection(suggested by procalcitonin), *n* (%)	6 (15.00%)	13 (32.50%)	0.048∗
Chest X-ray and CT findings			
Unilateral pneumonia, *n* (%)	2 (5.00%)	0 (0.00%)	0.82
Bilateral pneumonia, *n* (%)	38 (95.00%)	40 (100.00%)	0.40
No abnormal density shadow	0 (0.00%)		

^∗^ *P* values indicate differences between T2D patients and NDM patients. *P* < .05 was considered statistically significant.

**Table 3:** Laboratory findings of CBC analysis of patients with and without T2D infected with COVID-19.

Variables	Normal range	NDM group (*n* = 40)			T2D group (*n* = 40)			*P* value
		Median (IQR)	Increased No. (%)	Decreased No. (%)	Median (IQR)	Increased No. (%)	Decreased No. (%)	
RBC (×109/L)	3.9-5.1	4.04	0 (0.00%)	19 (47.50%)	4.14	0 (0.00%)	17 (42.50%)	0.51
Hb (g/L)	115-150	124.60	0 (0.00%)	16 (40.00%)	128.80	0 (0.00%)	12 (30.00%)	0.28
WBC (×10^9^/L)	3.5-9.5	5.36	0 (0.00%)	6 (15.00%)	7.22	6 (15.0%)	4 (10.00%)	0.01∗
NEUT (×109/L)	1.8-6.3	3.28	2 (5.00%)	3 (7.50%)	5.41	10 (25.00%)	1 (2.50%)	<0.01∗
LYMPH (×10^9^/L)	1.1-3.2	1.43	0 (0.00%)	17 (42.50%)	1.22	3 (7.50%)	18 (45.00%)	0.18
MONO (×10^9^/L)	0.1-0.6	0.50	6 (15.00%)	1 (2.50%)	0.52	9 (22.50%)	0 (0.00%)	0.76
PLT (×109/L)	125-350	226.30	1 (2.50%)	1 (2.50%)	226.30	2 (5.00%)	5 (12.50%)	0.99
CRP(mg/L)	>5	25.11	20 (50.00%)	0 (0.00%)	51.44	25 (62.50%)	0 (0.00%)	0.02∗
AST (U/L)	7-40	38.00	18 (45.00%)	1 (2.50%)	37.12	9 (22.50%)	0 (0.00%)	0.22
ALT (U/L)	13-35	32.20	12 (30.00%)	3 (7.50)	26.73	1 (2.50%)	2 (5.00%)	0.72
ALP (U/L)	50-135	78.30	2 (5.00%)	0 (0.00%)	72.20	1 (2.50%)	5 (12.50%)	0.63
GGT (U/L)	7-45	43.60	13 (32.50%)	0 (0.00%)	48.20	9 (22.50%)	0 (0.00%)	0.37
TP (g/L)	65-85	63.60	0 (0.00%)	25 (62.50%)	62.90	0 (0.00%)	25 (62.50%)	0.98
ALB (g/L)	40-55	37.60	0 (0.00%)	25 (62.50%)	35.60	0 (0.00%)	23 (57.50%)	0.08
GLB (g/L)	20-40	26.30	1 (2.50%)	4 (10.00%)	27.20	0 (0.00%)	2 (5.00%)	0.08
ALB/GLB	1.2-2.4	1.49	1 (2.50%)	3 (7.50%)	1.33	0 (0.00%)	7 (17.50%)	<0.01∗
TBIL (*μ*mol/L)	0-23	3.78	1 (2.50%)	0 (0.00%)	11.2	2 (5.00%)	0 (0.00%)	0.71
Urea (mmol/L)	0-8	17.40	2 (5.00%)	4 (10%)	5.30	2 (5.00%)	1 (2.50%)	0.36
Cr (*μ*mol/L)	2.6-7.5	66.30	3 (7.50%)	6 (15.00%)	60.30	3 (7.50%)	7 (17.50%)	0.50
UA (*μ*mol/L)	155-357	322	7 (17.50%)	6 (15.00%)	250	4 (10.00%)	0 (0.00%)	0.01∗
GLU (mmol/L)	3.9-6.1	5.04	0 (0.00%)	0 (0.00%)	9.60	40 (100%)	0 (0.00%)	<0.01∗
Lac (mmol/L)	0.5-1.5	2.45	20 (50.00%)	0 (0.00%)	2.99	30 (75.00%)	0 (0.00%)	0.02
K+ (mmol/L)	3.5-5.3	4.07	0 (0.00%)	6 (15.00%)	4.12	1 (2.50%)	7 (17.50%))	0.83
Na+(mmol/L)	137-147	141	4 (10.00%)	4 (10.00%)	140	1 (2.50%)	10 (25.00%)	0.01∗
Cl- (mmol/L)	99-110	105	3 (7.50%)	0 (0.00%)	104	0 (0.00%)	6 (15.00%)	<0.01∗
Ca2+ (mmol/L)	2.11-2.52	2.17	0 (0.00%)	11 (27.50%)	2.12	0 (0.00%)	22 (55.00%)	0.04∗
Correct Ca2+(mmol/g)	2.11-2.52	2.31	0 (0.00%)	1 (2.50%)	2.25	0 (0.00%)	4 (25.00%)	0.02∗
AGPK (mmol/L)	12-20	14.08	2 (5.0%)	3 (7.50%)	15.40	2 (5.00%)	6 (15.00%)	0..81
OSMO (mosm/L)	280-310	282.30	2 (5.00%)	8 (20.00%)	282	3 (7.50%)	8 (20.00%)	0.92
TCH (mmol/L)	<5.2	4.06	1 (2.50%)	0 (0.00%)	3.96	2 (5.00%)	0 (0.00%)	0.68
TG (mmol/L)	<1.7	1.54	7 (17.50%)	0 (0.00%)	1.75	11 (27.50%)	1 (2.50%)	0.27
LDL (mmol/L)	≥1	1.08	0 (0.00%)	17 (42.50%)	0.98	0 (0.00%)	20 (50.00%)	0.51
HDL (mmol/L)	<3.4	2.95	2 (5.00%)	1 (2.50%)	2.44	5 (12.50%)	0 (0.00%)	0.73

RBC: red blood cells count; Hb: hemoglobin; LYMPH: lymphocyte count; MONO: monocyte count; WBC: white blood cell count; NEUT: neutrophil count; PLT: platelet count; Hs-CRP: high-sensitivity C-reactive protein; CRP: C-reactive protein; ALT: alanine aminotransferase; AST: aspartate aminotransferase; ALP: alkaline phosphatase; GGT: *γ*-glutamyltransferase; TP: total protein; ALB: albumin; GLB: globulin; TBIL: total bilirubin; Cr: creatinine; UA: uric acid; GLU: glucose, Lac: lactic acid; AGPK: anion gap; OSMO: osmotic pressure; TCH: total cholesterol; TG: triacylglycerol; LDL: low-density lipoprotein cholesterol; HDL: high-density lipoprotein cholesterol. ^∗^*P* values indicate differences between T2D patients and NDM patients. *P* <0.05 was considered statistically significant.

**Table 4:** Laboratory findings of the coagulation function of patients with and without T2D infected with COVID-19.

Variables	Normal range	NDM group (*n* = 39)			T2D group (*n* = 30)			*P* value
		Median (IQR)	Increased No. (%)	Decreased No. (%)	Median (IQR)	Increased No. (%)	Decreased No. (%)	
Prothrombin time (sec)	9-13	11.55	2 (5.13%)	0 (0.00%)	11.71	0 (0.00%)	0 (0.00%)	0.82
PT activity (%)	75-135	93.60	0 (0.00%)	8 (20.51%)	89.74	0 (0.00%)	1 (3.33%)	0.54
PT international normalized ratio	0.76-1.24	0.98	3 (7.69%)	4 (10.26%)	1.41	2 (6.67%)	5 (16.67%)	0.24
Activated partial thrombin time (sec)	25-31.3	27.83	4 (10.26%)	1 (2.56%)	26.96	3 (10.0%)	0 (25%)	0.16
Thrombin time (sec)	14-21	18.02	2 (5.13%)	0 (0.00%)	18.21	1 (3.33%)	0 (0.00%)	0.52
Fibrinogen (g/L)	2-4	4.26	15 (38.46%)	0 (0.00%)	4.05	16 (53.30%)	1 (3.33%)	0.75
D-dimer (mg/L)	0-0.55	2.20	24 (61.54%)	0 (0.00%)	3.19	22 (73.33%)	0 (0.00%)	0.17
Fibrinogen degradation products (mg/L)	0-5	7.56	14 (35.90%)	0 (0.00%)	10.25	8 (26.67%)	0 (0.00%)	0.36
Antithrombin III activity (%)	80-120	89.29	1 (2.56%)	8 (20.51%)	86.86	0 (0.00%)	6 (20.0%)	0.72

## Data Availability

The data that support of this study are available from the corresponding author upon reasonable request.

## Supplementary Materials

Supplementary 1 Figure 1. Representative data of a shorter infection and hospitalization timeline from illness onset to hospital treatment for COVID-19 patients with and without T2D.

Supplementary 2 Figure 2. Representative data of chest CT images of COVID-19 patients with and without T2D.
