# Supplementary material for: Differences in the Clinical and Hematological Characteristics of COVID-19 Patients with and without Type 2 Diabetes
Source: J Diabetes Res. 2020 Dec 2;2020:1038585. doi: 10.1155/2020/1038585 (PMC7745050; doi:10.1155/2020/1038585)
Supplement: Supplementary 1 — Figure 1. Representative data of a shorter infection and hospitalization timeline from illness onset to hospital treatment for COVID-19 patients with and without T2D. [file 1038585.f1.pptx]

## Slide 1
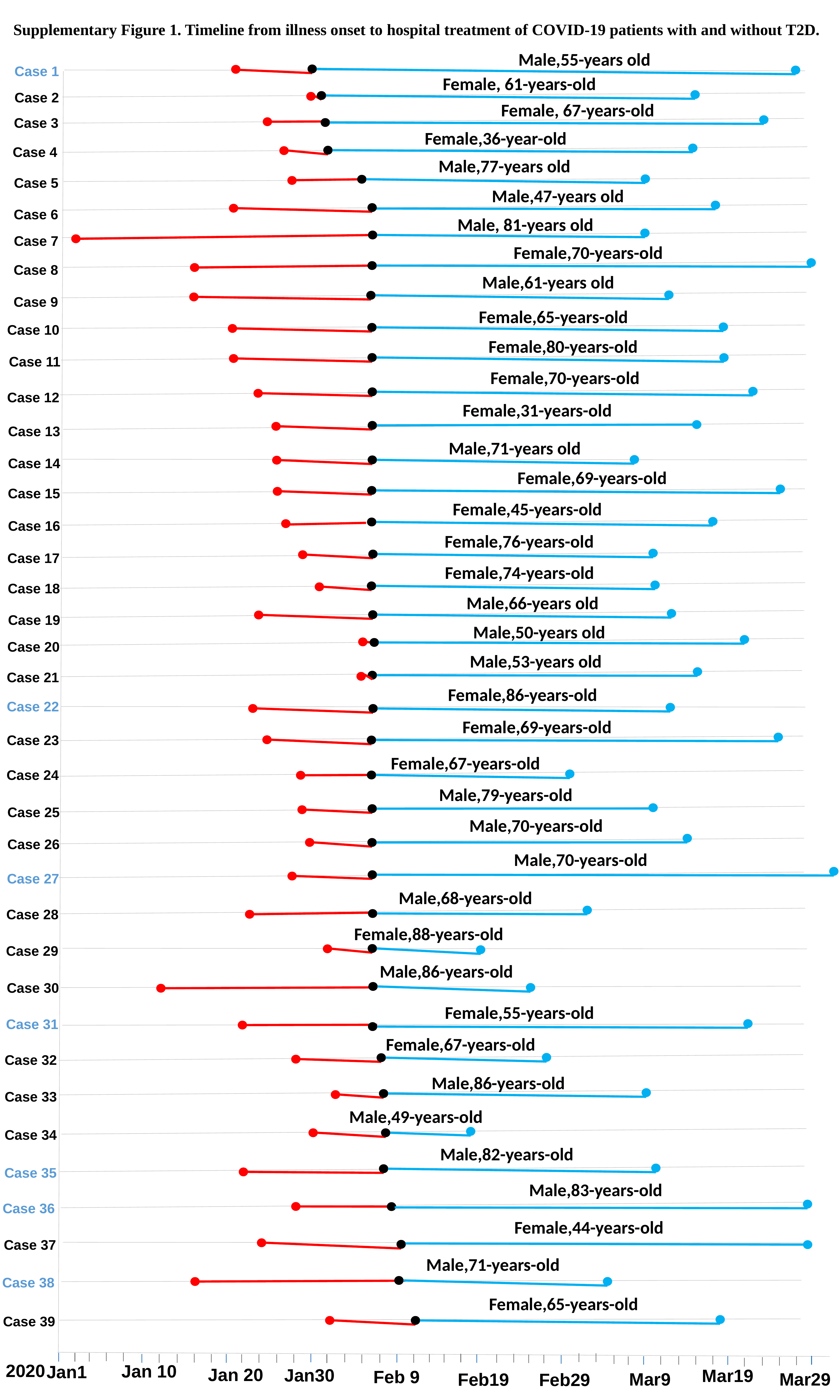

Supplementary Figure 1. Timeline from illness onset to hospital treatment of COVID-19 patients with and without T2D.
Male,55-years old
Case 1
Female, 61-years-old
Case 2
Female, 67-years-old
Case 3
Female,36-year-old
Case 4
Male,77-years old
Case 5
Male,47-years old
Case 6
Male, 81-years old
Case 7
Female,70-years-old
Case 8
Male,61-years old
Case 9
Female,65-years-old
Case 10
Female,80-years-old
Case 11
Female,70-years-old
Case 12
Female,31-years-old
Case 13
Male,71-years old
Case 14
Female,69-years-old
Case 15
Female,45-years-old
Case 16
Female,76-years-old
Case 17
Female,74-years-old
Case 18
Male,66-years old
Case 19
Male,50-years old
Case 20
Male,53-years old
Case 21
Female,86-years-old
Case 22
Female,69-years-old
Case 23
Female,67-years-old
Case 24
Male,79-years-old
Case 25
Male,70-years-old
Case 26
Male,70-years-old
Case 27
Male,68-years-old
Case 28
Female,88-years-old
Case 29
Male,86-years-old
Case 30
Female,55-years-old
Case 31
Female,67-years-old
Case 32
Male,86-years-old
Case 33
Male,49-years-old
Case 34
Male,82-years-old
Case 35
Male,83-years-old
Case 36
Female,44-years-old
Case 37
Male,71-years-old
Case 38
Female,65-years-old
Case 39
2020
Jan 10
Jan1
Jan 20
Jan30
Mar19
 Feb 9
Mar9
Mar29
 Feb19
 Feb29
